# Methamphetamine causes acute hyperthermia-dependent liver damage

**DOI:** 10.1002/prp2.8

**Published:** 2013-10-01

**Authors:** Laura E Halpin, William T Gunning, Bryan K Yamamoto

**Affiliations:** 1 Department of Neurosciences, University of Toledo College of Medicine3000 Arlington Ave., Toledo, Ohio, 43614; 2 Department of Pathology, University of Toledo College of Medicine3000 Arlington Ave., Toledo, Ohio, 43614

**Keywords:** Ammonia, hyperthermia, liver damage, methamphetamine

## Abstract

Methamphetamine-induced neurotoxicity has been correlated with damage to the liver but this damage has not been extensively characterized. Moreover, the mechanism by which the drug contributes to liver damage is unknown. This study characterizes the hepatocellular toxicity of methamphetamine and examines if hyperthermia contributes to this liver damage. Livers from methamphetamine-treated rats were examined using electron microscopy and hematoxylin and eosin staining. Methamphetamine increased glycogen stores, mitochondrial aggregation, microvesicular lipid, and hydropic change. These changes were diffuse throughout the hepatic lobule, as evidenced by a lack of hematoxylin and eosin staining. To confirm if these changes were indicative of damage, serum aspartate and alanine aminotransferase were measured. The functional significance of methamphetamine-induced liver damage was also examined by measuring plasma ammonia. To examine the contribution of hyperthermia to this damage, methamphetamine-treated rats were cooled during and after drug treatment by cooling their external environment. Serum aspartate and alanine aminotransferase, as well as plasma ammonia were increased concurrently with these morphologic changes and were prevented when methamphetamine-induced hyperthermia was blocked. These findings support that methamphetamine produces changes in hepatocellular morphology and damage persisting for at least 24 h after drug exposure. At this same time point, methamphetamine treatment significantly increases plasma ammonia concentrations, consistent with impaired ammonia metabolism and functional liver damage. Methamphetamine-induced hyperthermia contributes significantly to the persistent liver damage and increases in peripheral ammonia produced by the drug.

## Introduction

Methamphetamine (METH) is a widely abused psychostimulant that causes persistent damage to dopamine and serotonin terminals (Ricaurte et al. 1980; Seiden et al. 1988), yet few studies have examined peripheral organs for damage caused by the drug. Many studies of METH focus on the central nervous system effects of the drug, as the drug contributes to altered neuronal function, addiction, and cellular damage. However, damage to the liver and other organs have also been reported after METH exposure (Smith and Fischer 1970; Kamijo et al. 2002; Wijetunga et al. 2003; Ago et al. 2006). Recently, a connection between METH-induced liver damage, increased peripheral and brain ammonia, and long-term dopamine and serotonin depletions caused by the drug have been established, thus highlighting the significance of peripheral organ damage in mediating the neurotoxicity of METH (Halpin and Yamamoto 2012). Despite these findings, the hepatocellular damage produced by METH has not been extensively characterized in vivo. Further characterization and understanding of the hepatotoxicity produced by METH is significant because this hepatotoxicity appears to contribute to its well-established neurotoxicity.

METH-induced hepatocellular damage may be the consequence of the direct effects of the drug on the liver, other peripheral effects on cardiac function, or alterations in body temperature. In fact, METH causes considerable hyperthermia which is strongly linked to the neuronal damage (Bowyer et al. 1992, 1994; Albers and Sonsalla 1995; Haughey et al. 2000; Xie et al. 2000; Kiyatkin and Sharma 2009). The significant hyperthermia (39–42°C) produced by amphetamine exposure likely results from drug-induced increases in monoamine concentrations and the complex integration of the subsequent hyperlocomotion, altered metabolism, changes in hypothalamic neurotransmission, and vasoconstriction (Wang et al. 1990; Brown et al. 2007; Sprague et al. 2007; Benamar et al. 2008). Also worth noting is that hyperthermia, in the context of heat stroke, causes changes in hepatocellular morphology reminiscent of that observed 24 h after acute METH exposure (Kew et al. 1970; Bianchi et al. 1972; Weigand et al. 2007; Halpin and Yamamoto 2012). Recently, in vitro studies have shown that high concentrations of METH and other amphetamines synergize with increases in exogenous temperature to produce damage to hepatocytes indicative of both apoptosis and necrosis and further support a link between METH-induced hyperthermia and liver damage (da Silva et al. 2013a,b). Hyperthermia contributes to significant cellular damage and death in a manner that is dependent on both the amount and duration of temperature elevation (Palzer and Heidelberger 1973; Sapareto et al. 1978). The damage produced by hyperthermia results from the effects of increased temperature on protein unfolding and cell cycle arrest (Westra and Dewey 1971; Harisiadis et al. 1977; Landry et al. 1982). Accordingly, if METH-induced hyperthermia plays a significant role in the liver damage and increases in ammonia observed during drug exposure, this may represent a mechanism by which hyperthermia contributes significantly to the neurotoxicity of METH.

Beyond understanding the mechanism of METH-induced structural hepatocellular damage, it is also important to determine if there is a correlation between morphologic alteration and diminished liver function. A key function of the liver is the metabolism of ammonia to urea via the urea cycle. When liver function is compromised, ammonia accumulates and has neurotoxic consequences (Felipo and Butterworth 2002). Along these lines, it has been demonstrated that the increases in peripheral and brain ammonia are concurrent with METH-induced hyperthermia and liver damage and contribute to the neuronal damage characteristic of METH (Halpin and Yamamoto 2012). Accordingly, it is important to examine if the prevention of METH-induced liver damage also blocks the increases in peripheral ammonia because this represents a marker of liver function and the neurotoxicity produced by METH.

Changes in hepatocellular morphology can be detected using hematoxylin and eosin staining for acidic and basic components of the tissue at the light microscopic level. In addition, specific ultrastructural changes to hepatocytes can be characterized using electron microscopy (Katz 1989; Batt and Ferrari 1995). Hepatocellular enzyme serum concentrations can also be utilized as selective biomarkers for hepatic tissue damage (Ozer et al. 2008). Therefore, to further characterize acute liver damage after a binge dosing regimen of METH, we used transmission electron microscopy to assess damage at 24 h after METH. We have also quantified AST and ALT as well as peripheral ammonia at this same time point to examine if METH produces increases in these measures that persist for 24 h after drug exposure. The rationale for examining the contribution of METH-induced hyperthermia to these effects is based upon two primary considerations: (1) the strong similarity of hepatocellular changes after METH with other hyperthermic stimuli and (2) the observation that hyperthermia is a complication of acute METH exposure which can be effectively managed in patients in acute care settings.

## Materials and Methods

### Animal treatment

Male Sprague Dawley rats were treated with METH (2, 5, or 10 mg/kg i.p. every 2 h ×4) or saline (1 mL/kg i.p. every 2 h ×4). This dose simulates the concentration and binge dosing paradigm reported in METH-dependent humans and produces the same long-term neuronal damage seen in humans after METH exposure (McCann et al. 1998; Volkow et al. 2001; McKetin et al. 2006; Cruickshank and Dyer 2009). The examination of the hepatocellular effects of this neurotoxic dose of METH is significant for further understanding the hepatic damage and increases in ammonia which have been shown mediate to the neurotoxicity of the drug (Halpin and Yamamoto 2012). Rats were killed by rapid decapitation 24 h after the last injection of drug or were anesthetized for ventricular perfusion and exsanguination for ultrastructural investigation of tissues. Body temperature was monitored during the experiments using transponders that were subcutaneously implanted 2 days before drug treatment (IPTT-300 transponder, BDMS, Seaford, DE). Implantation of temperature transponders allowed for remote, repeated, and noninvasive monitoring of temperature during drug treatment. All experiments were carried out at an ambient temperature of 21–23°C. Core temperature was measured in all rats during drug treatment at an hour after each injection. In some experiments, METH-induced hyperthermia was blocked (Figs. 3–6; METH-cooled group) by cooling the external environment with ice packs placed around the test cages. Temperature was also measured in these rats every 10 min throughout drug treatment. This was done in all groups to ensure that the body temperature of METH-treated normothermic rats was kept at the same temperature as saline-treated rats. The last measurement was recorded at 5 h after the last injection and occurred just prior to the beginning of the dark cycle of rats. All treatments were carried out in accordance with the National Institute of Health Guide for Care and Use of Rats. All treatments have also been approved by the University of Toledo Institutional Animal Care and Use Committee.

### Transmission electron microscopy

Twenty-four hours after drug treatment, rats were anesthetized (5 mg/kg of xylazine and 75 mg/kg of ketamine) and transcardially perfused via the left ventricle with 100 mL of phosphate buffered saline (pH 7.4) subsequently followed by 200 mL of 3% glutaraldehyde buffered with 0.2 mol/L sodium cacodylate (pH 7.2). After perfusion, livers were harvested and processed using standard electron microscopy procedures including fixation with 1% osmium tetroxide followed by an en bloc stain using saturated aqueous uranyl acetate (pH 3.3), and dehydrated using a series of gradient ethanol concentrations (30%, 50%, 70%, 90%, 95%, and 100%) and acetone. A combination of acetone and resin Embed It™ Low Viscosity Resin Kit, Polysciences Inc. (Warrington, PA) was used to infiltrate the tissue with resin. Tissue was then embedded in 100% resin and polymerized overnight at 80°C. Ultra thin sections (60–90 nm) were acquired using an OMU3 C. Reichert Ultramicrotome and stained using saturated uranyl acetate and Reynold's lead citrate. Images were acquired using a Philips CM 10 transmission electron microscope (FEI, Hillsboro, OR).

### Hematoxylin and eosin staining

Liver tissue was postfixed overnight in 10% buffered formalin (Fisher Scientific, Pittsburgh, PA) and embedded in paraffin. Blocks were sectioned at 4 μm and stained with hematoxylin and eosin using a Leica Autostainer XL (Buffalo Grove, IL). Sections were reviewed by a pathologist who was blinded to treatment group.

### Aspartate aminotransferase, alanine aminotransferase, and plasma ammonia determination

Trunk blood was collected after rapid decapitation for measurement of serum aspartate aminotransferase (AST) and alanine aminotransferase (ALT) and plasma ammonia. Serum was prepared by allowing blood to coagulate and then centrifuged for 10 min at 3000*g* to separate the serum. Plasma was prepared by collecting blood in BD Microtainer Plasma Separation Tubes (Becton, Dickenson and Company, Franklin Lakes, NJ) and centrifuging for 2 min at 10,000*g*. AST and ALT levels and ammonia concentration was determined using a UniCel DxC800 Synchron Clinical System (Beckman Coulter, Brea, CA).

### Statistical methods

Analysis of AST and ALT levels and ammonia concentrations in the three different treatment groups was carried out using a one-way ANOVA with post hoc Tukey tests to determine differences between groups. These tests were performed using SigmaPlot 11.0 Software (Systat Software, Inc. SigmaPlot for Windows; San Jose, CA). All data are presented as Mean ± SEM. Sample sizes were determined based on a power of 80% or greater and α level in all studies was 0.05 or less.

## Results

### METH and ultrastructural liver damage

**Figure 1:**
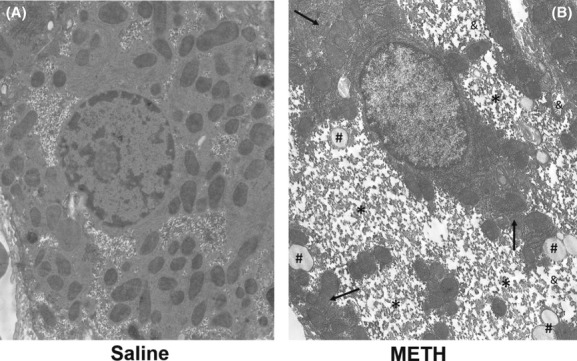
Ultrastructural changes in liver 24 h after methamphetamine exposure. Representative images from METH- or saline-treated groups are shown. Hepatocytes of METH-treated rats have increased microvesicular lipid (#), hydropic change (&), increased intracellular glycogen stores (*), and mitochondrial aggregation (arrow) compared to control animals (5200× magnification).

Transmission electron microscopy was used to characterize the METH-induced alterations in hepatocellular morphology on an ultrastructural level. Livers were examined 24 h after treatment with METH or saline. Ultrastructural changes seen in hepatocytes of 100% of METH-treated rats include microvesicular lipid, mild hydropic change, increased cytoplasmic glycogen, and mitochondrial aggregation (Fig. 1).

### Dose dependence of METH on hyperthermia and alterations in 24-h hepatocellular morphology

**Figure 2:**
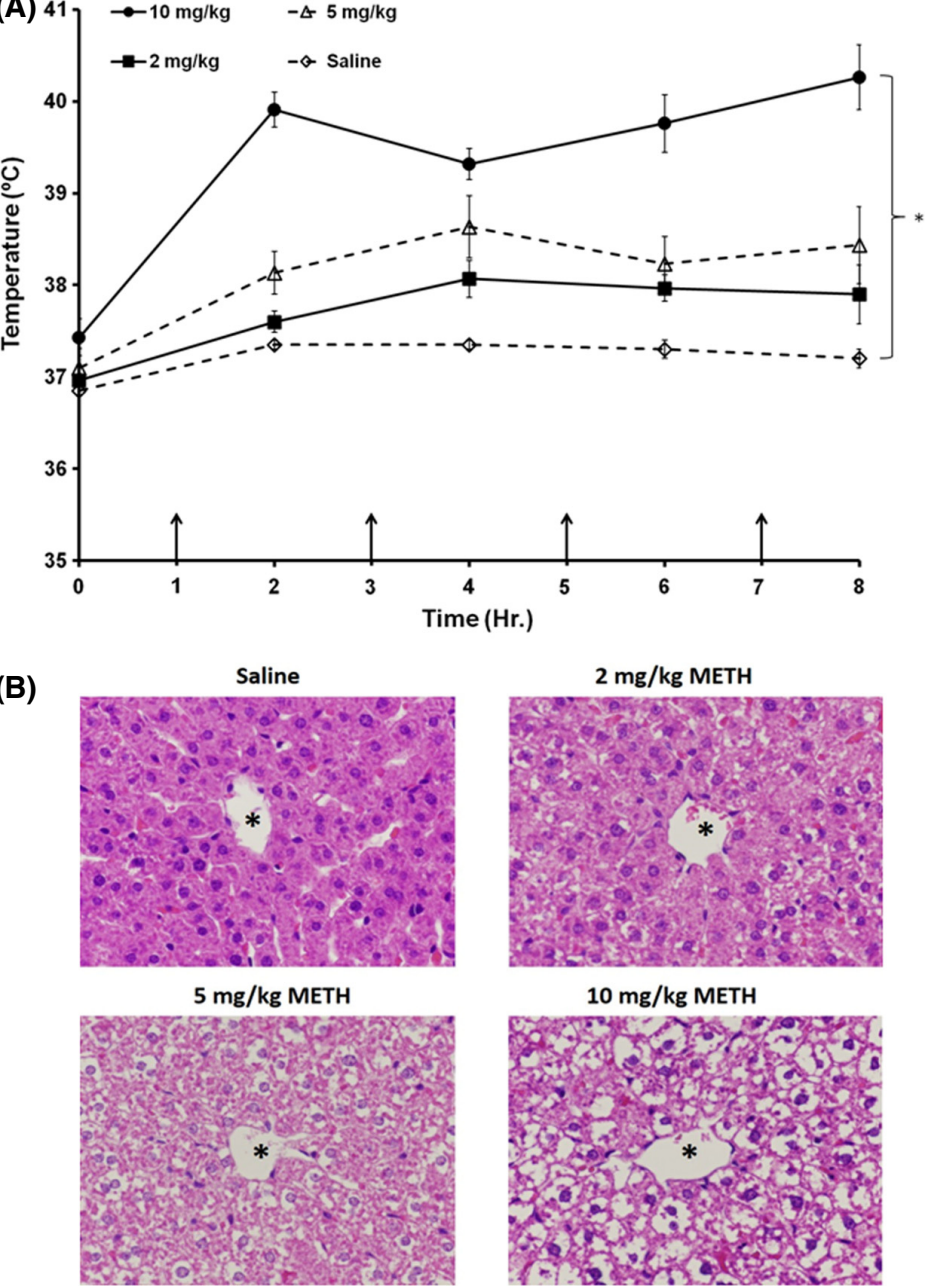
Effect of METH dose on hyperthermia and alterations in hepatocellular morphology. Rats were treated with 2, 5, and 10 mg/kg of METH ×4 injections, every 2 h or saline. (A) During drug treatment temperature was measured and each larger dose of METH produced a trend toward increased hyperthermia throughout treatment. Treatment with 10 mg/kg METH produces significant hyperthermia throughout drug treatment. (B) When changes in hepatocellular morphology were examined 24 h later, increases in METH dose also appeared to dose dependently decrease hepatocellular cytoplasmic staining. *P < 0.05, (*n* = 3–5 per group).

To examine if the METH-induced hyperthermia and the changes in hepatocellular morphology produced by METH were affected differentially by the dose of METH, rats were treated with either saline or 2, 5, or 10 mg/kg of METH, every injection administered every 2 h for a total of four injections. We have shown that METH produces alterations in hepatocellular morphology at 24 h after drug exposure, however, prior to these studies, it was unknown if these changes were dose dependent (Halpin and Yamamoto 2012). During drug treatment, temperature was measured and each larger dose of METH produced a trend toward increased hyperthermia throughout treatment (Fig. 2A). Treatment with 10 mg/kg METH produced significant increases in hyperthermia throughout treatment. When changes in hepatocellular morphology were examined 24 h later, increases in METH dose also appeared to dose dependently decrease hepatocellular cytoplasmic staining (Fig. 2B).

### Environmental cooling and methamphetamine-induced hyperthermia

**Figure 3:**
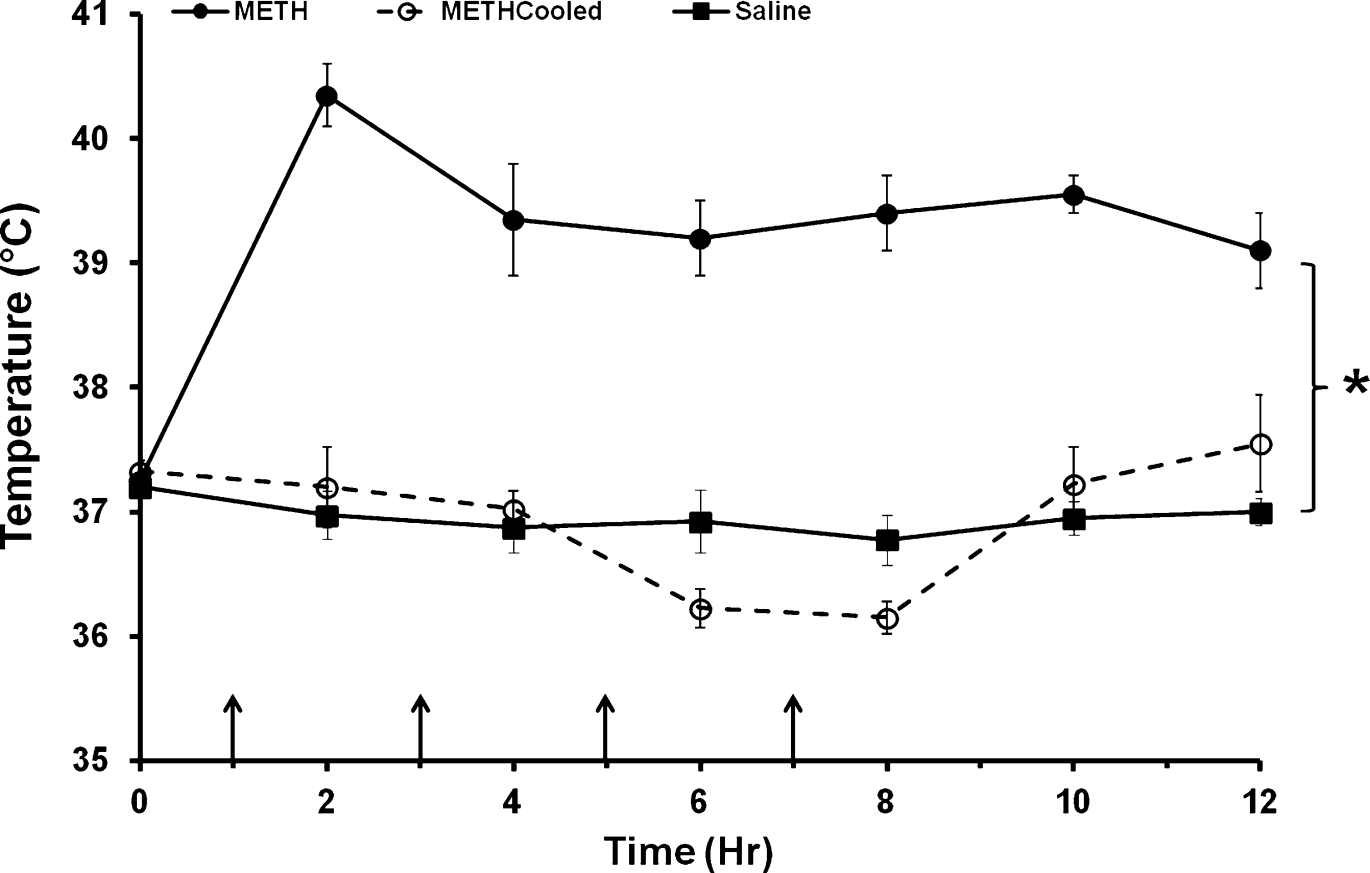
Effect of environmental cooling on methamphetamine-induced hyperthermia. Rats were treated with METH or saline (arrows denote injections). The external environment of a group of METH-treated rats was cooled throughout drug treatment and for 5 h after the last injection to prevent METH-induced hyperthermia. Cooling was regulated to ensure that the body temperatures of METH-treated cooled rats were similar to that of saline-treated rats. METH produced a significant elevation in body temperature throughout treatment compared to the saline-treated and METH-cooled groups. Body temperature of METH-cooled rats did not differ significantly from saline-treated rats. *P < 0.05 (*n* = 10 per group).

To examine if METH-induced hyperthermia contributes to the liver damage observed 24 h after exposure to METH, METH-induced acute hyperthermia was prevented by cooling METH-treated rats to the body temperatures of saline control rats throughout METH treatment and for 5 h after the end of drug treatment, until the beginning of the dark cycle of the rats. METH treatment caused significant hyperthermia throughout and after METH treatment, which was fully blocked by cooling the external environment of rats (Fig. 3). Cooling resulted in METH-treated rats with normothermic body temperatures.

### Hyperthermia and METH-induced changes in liver morphology

**Figure 4:**
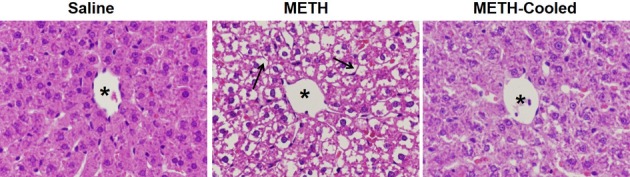
Effect of hyperthermia on methamphetamine-induced changes in liver morphology. Hepatocellular morphology was evaluated using H&E sections from livers collected at 24 h after the last drug injection. A representative picture from all groups of the hepatic lobule near the central vein is shown (asterisk denotes central vein). The livers of METH-treated rats show less hepatocellular cytoplasmic staining (arrows denote cytoplasm) compared to saline-treated rats. The cytoplasmic clearing was prevented when METH-induced hyperthermia was blocked by cooling the rats to normothermic temperatures.

Hematoxylin and eosin staining was used to determine if hyperthermia contributes to the alterations in hepatocellular morphology throughout the hepatic lobule occurring 24 h after METH in hyperthermic, normothermic, and saline control rats. At 24 h after METH exposure, there was considerable clearing of cytoplasmic staining in hepatocytes throughout the hepatic lobule, comparable to that which had been demonstrated after METH treatment (Halpin and Yamamoto 2012). The prevention of METH-induced hyperthermia blocked the changes in cellular morphology seen in the livers of METH-treated rats (Fig. 4). The cellular alterations in hyperthermic METH-treated rats were evident in 100% of the METH-treated rats and were prevented in all normothermic METH-treated rats.

### Hyperthermia and METH-induced hepatocellular damage

To examine if the changes observed in hepatocellular morphology at 24 h after drug treatment are indicative of cellular damage and if hyperthermia contributes to METH-induced cellular damage, serum concentrations of the hepatocellular enzymes AST and ALT (Ozer et al. 2008) were measured 24 h after treatment with METH in rats that were hyperthermic, cooled to normothermia, or saline control rats. Serum AST concentration was 335.5 ± 29.1 IU/L in hyperthermic METH-treated rats and 177.4 ± 13.3 IU/L in saline-treated rats, representing an increase of 89.1 ± 16.4% in hyperthermic METH-treated rats. In normothermic METH-treated rats, AST levels were 231.9 ± 20.4 IU/L, representing an increase of only 30.7 ± 11.5%, compared to saline-treated rats. A one-way ANOVA revealed significant differences between the three groups (F_(2, 29)_ = 13.395, *P* < 0.001). A post hoc Tukey test showed significant differences in serum AST levels in hyperthermic METH- versus saline-treated rats (q = 7.205, *P* < 0.001), and hyperthermic METH- versus normothermic METH-treated rats (q = 4.721, *P* < 0.05); however, there was no significance differences in serum AST in saline-treated versus normothermic METH-treated rats (q = 2.484, *P* = 0.203).

ALT concentration was 83.9 ± 6.3 IU/L in hyperthermic METH-treated rats and 67.5 ± 2.6 IU/L in saline-treated rats, representing a increase of 24.3 ± 9.3% in METH-treated rats. In normothermic METH-treated rats, ALT levels were 64.8 ± 2.8 IU/L. A one-way ANOVA revealed significant differences between the three groups (F_(2, 29)_ = 10.005, *P* < 0.001). A post hoc Tukey test revealed significant differences in serum ALT levels in hyperthermic METH- versus saline-treated rats (q = 5.122, *P* < 0.05), and hyperthermic METH- versus normothermic METH-treated rats (q = 5.800, *P* < 0.05), however, there was no significance difference between saline-treated and normothermic METH-treated rats (q = 0.678, *P* = 0.882).

### Effect of hyperthermia on METH-induced increases in plasma ammonia

To examine if METH-induced increases in plasma ammonia persist for 24 h and if hyperthermia contributes to those increases, plasma ammonia was measured 24 h after treatment with METH in hyperthermic and normothermic rats, and saline controls. Plasma ammonia concentration was 154.17 ± 7.51 μmol/L in hyperthermic METH-treated rats and 71.78 ± 6.51 μmol/L in saline-treated rats, representing an increase of 114.8 ± 10.5%. In normothermic METH-treated rats, plasma ammonia concentration was 80.50 ± 6.02 μmol/L, representing only a 12.2 ± 8.3% increase, compared with saline-treated rats. A one-way ANOVA revealed significant differences between the three groups (F_(2, 24)_ = 38.45, *P* < 0.001). A post hoc Tukey test examining group differences revealed significant differences in plasma ammonia in hyperthermic METH- versus saline-treated rats (t = 9.198, *P* < 0.001) and hyperthermic METH versus normothermic METH rats (t = 7.481, *P* < 0.001), however, there was no significant difference in plasma ammonia in saline-treated versus METH normothermic rats (t = 0.996, *P* = 0.330).

## Discussion

This study characterized METH-induced hepatocellular damage and examined if METH-induced hyperthermia contributes significantly to both structural liver damage and functional consequences associated with hepatotoxicity. METH produced changes in hepatocellular ultrastructure indicative of generalized cellular stress and damage at 24 h after drug exposure. Consistent with these ultrastructural changes, METH treatment induced an extensive decrease in hepatocellular cytoplasmic H&E staining. Increases in serum AST and ALT at this same time point confirmed that there was cellular damage concurrent with these structural changes. To determine if METH-induced hyperthermia contributes to this hepatocellular damage, hyperthermia was prevented in rats treated with METH by cooling the external environment. The prevention of hyperthermia in METH-treated rats blocked METH-induced changes in hepatocellular morphology, as well as increases in AST and ALT, suggesting that hyperthermia plays a significant role in METH-induced liver damage. At this same time point, METH also increases plasma ammonia levels and as with the METH-induced liver damage, these increases in peripheral ammonia were blocked by preventing hyperthermia.

We recently reported that METH-induced liver damage was associated with increases in brain and peripheral ammonia and long-term decreases in brain dopamine and serotonin content, but the mechanisms by which METH causes hepatocellular changes were unknown (Halpin and Yamamoto 2012). Electron microscopy of the liver after METH shows microvesicular fatty and hydropic change, increased intracellular glycogen and mitochondrial aggregation (Fig. 1). These changes are consistent with the generalized hepatocellular damage reported after exposure to hyperthermia and/or heatstroke (Kew et al. 1970; Weigand et al. 2007) evidenced by increases in electron-lucent vacuoles along the sinusoidal border that are indicative of membrane damage and alterations in mitochondria (Wills et al. 1976; Kew et al. 1978). Aggregation of mitochondria may indicate mitochondrial dysfunction and cellular damage (Haga et al. 2003, 2005). Dysfunction of hepatocellular mitochondria may contribute significantly to increases in ammonia as carbamoylphosphate synthetase-1 and ornithine transcarbamylase are two key enzymes in the urea cycle, located within mitochondria (Adeva et al. 2012). In addition to these markers of damage, METH also increases intracellular glycogen. This may be representative of increased glycogen formation or decreased glycogen breakdown after METH exposure. It is unclear if this increase in glycogen is a physiologic or pathologic response to METH treatment. Glycogen is made in the liver as a form of energy storage, increases postprandially, and is regulated by insulin (Parkes and Grieninger 1985). Glycogen can also accumulate pathophysiologically in conditions of insulin insensitivity or after exposure to alkaloid compounds which inhibit glycogen breakdown (Saul et al. 1985; Messeri et al. 2012). Regardless, METH-induced increases in liver glycogen may represent significant METH-induced alterations in energy metabolism, hepatic function, and warrant further study.

To examine the extent of alterations in hepatocellular morphology, H&E staining was used to visualize if this damage is dose dependent and generalized throughout the hepatic lobule. Escalating doses of METH produced respective increases in hyperthermia and as well as decreases in hepatocellular staining. These findings suggest that METH produces dose-dependent damage to the liver, which appears to be mediated by hyperthermia (Fig. 2). A considerable decrease in H&E staining of the hepatocellular cytoplasm throughout the lobule was observed at 24 h after METH exposure (Fig. 4). This decrease in staining is a likely consequence of increased accumulation of hydrophobic substances in the cytoplasm. METH-induced alterations in hepatocellular ultrastructure (Fig. 1), including increases in glycogen, microvesicular lipid and hydropic change all represent increased accumulation of hydrophobic substances, which do not stain with the acidic and basic H&E staining. This extensive lack of H&E staining throughout the hepatic lobule in METH-treated rats demonstrates that the effects of METH on hepatocellular morphology are widespread.

**Figure 5:**
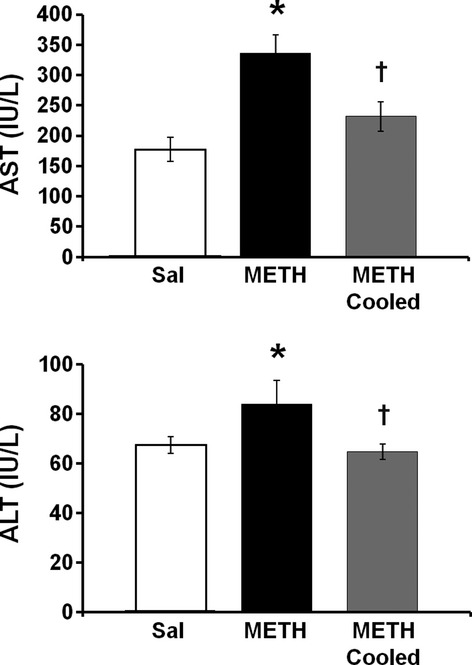
Effect of hyperthermia on methamphetamine-induced increases in serum aspartate aminotransferase (AST) and alanine aminotransferase (ALT). Serum AST and ALT were measured 24 h after treatment with METH or saline. A group of METH-treated rats were cooled during and after treatment to prevent METH-induced hyperthermia. (A) AST was significantly elevated by 89.1 ± 16.4% (mean ± SEM) in METH-treated rats compared to saline-treated rats and this increase was blocked when hyperthermia was prevented in METH-treated rats (METH cooled). (B) ALT was significantly elevated by 24.3 ± 9.3% (Mean ± SEM) in METH-treated rats compared to saline-treated rats. This increase was blocked when METH-induced hyperthermia was prevented (METH cooled). *P < 0.05 compared to saline-treated rats, †*P* < 0.05 compared to METH-treated rats (*n* = 10 per group).

To confirm if these changes in morphology are indicative of damage, we measured serum concentrations of AST and ALT. AST and ALT are hepatocellular enzymes that are released into the blood during cell damage and together with the increases in ammonia and altered hepatocellular morphology reported here, support that METH causes liver damage (Ozer et al. 2008). Increases in AST alone could signify generalized tissue damage including rhabdomyolysis, however, concurrent alterations in each of these variables support hepatocellular damage (Weibrecht et al. 2010). Other tissues that may be potentially damaged by METH may include the muscle, cardiac, and renal systems (Wijetunga et al. 2003; Ago et al. 2006). Both AST and ALT were significantly elevated at 24 h after METH treatment (Fig. 5) and support the conclusion that the changes in cellular morphology observed at this time point represent cellular damage. These findings also support the observation of acute liver damage previously reported at 2 and 24 h after METH treatment (Halpin and Yamamoto 2012) and suggest that METH is capable of producing hepatocellular damage that persists for 24 h after drug exposure, that in turn, contributes to the neurotoxicity of the drug.

We examined if METH hepatotoxicity was a result of METH-induced hyperthermia based on the fact that the alterations in hepatocellular morphology can be a response to hyperthermia. Amphetamines produce significant increases in body temperature as a result of alterations in both brain and peripheral physiology (Wang et al. 1990; Brown et al. 2007; Sprague et al. 2007; Benamar et al. 2008). The current findings show that when METH-induced hyperthermia is prevented, the changes and hepatocellular morphology and increases in AST and ALT seen 24 h after METH treatment are blocked (Figs. 3 and 4). These findings indicate that hyperthermia contributes significantly to the hepatotoxicity of the METH and extend previous findings that increases in body temperature play a significant role in METH-induced brain damage (Bowyer et al. 1993, 1994) and hepatocellular damage after the METH analog, 3,4-methylenedioxymethamphetamine (MDMA) (Carvalho et al. 2001).

The primary mechanism by which hyperthermia is believed to contribute to cellular damage is through oxidative stress, as evidenced by increases in lipid peroxidation and depletions in reduced glutathione after hyperthermia exposure (Skibba et al. 1991; Carvalho et al. 1997). Exogenous increases in temperature, combined with exposure to high concentrations of METH have been shown to contribute to damage to hepatocytes in vitro, further supporting a role for hyperthermia in METH hepatotoxicity (da Silva et al. 2013a,b). Although hyperthermia appears to be an essential component of METH hepatotoxicity, METH may contribute to hepatocellular damage though other mechanisms. METH has been shown to reach some of the highest and longest lasting concentrations in liver and accordingly, has the potential to be directly hepatotoxic (Volkow et al. 2010). METH is metabolized in the liver by the cytochrome p450 system (Lin et al. 1997) that can lead to the formation of oxidative byproducts and result in cellular damage (Moon et al. 2008; Pourahmad et al. 2010; Letelier et al. 2011). METH has also been shown to have extensive effects on cardiovascular function, causing both cardiac dysfunction and widespread vasospasm (Wang et al. 1990; Wijetunga et al. 2003; Chen 2007), both of which may impede hepatic blood flow, leading to ischemia and contributing to hepatotoxicity.

**Figure 6:**
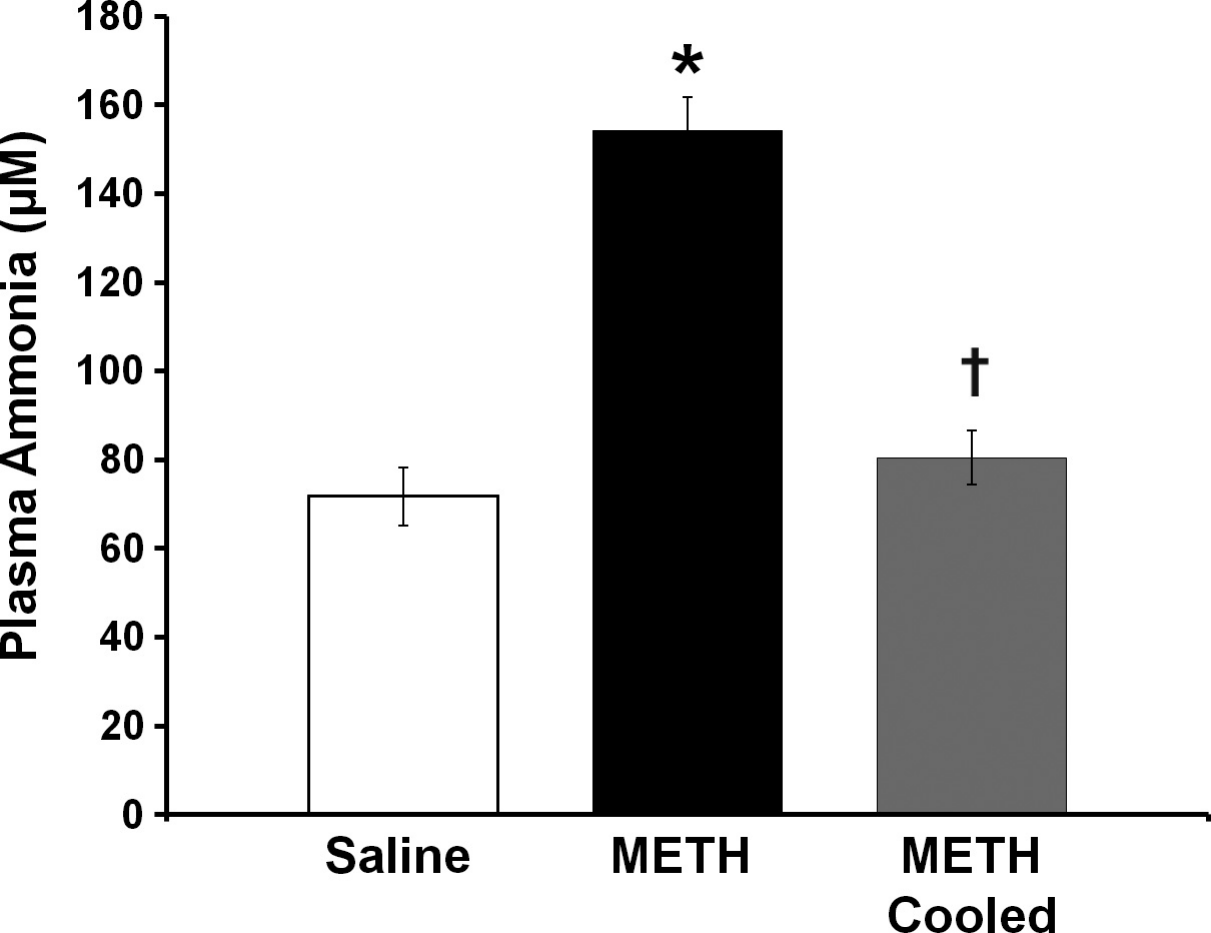
Effect of hyperthermia on methamphetamine-induced persistent increases in plasma ammonia. Plasma ammonia was measured 24 h after treatment with METH or saline. A group of METH-treated rats were cooled to prevent METH-induced hyperthermia. METH treatment significantly elevated plasma ammonia concentrations by 114.8 ± 10.5%. This increase was blocked by preventing METH-induced hyperthermia (METH cooled) *P < 0.05 compared to saline-treated rats, †*P* < 0.05 compared to METH-treated rats (*n* = 10 per group).

Peripheral ammonia levels were also measured at 24 h after METH exposure to determine if the hepatotoxicity at this time point had functional consequences. The liver is the primary organ of ammonia metabolism via the urea cycle. When liver function is compromised, ammonia accumulates and leads to neuronal damage (Felipo and Butterworth 2002). The measurement of ammonia in the context of METH toxicity is also significant as ammonia has been shown to be a key mediator of the neurotoxicity of the drug (Halpin and Yamamoto 2012). Plasma ammonia levels were significantly elevated at 24 h after METH exposure and this elevation was blocked by preventing METH-induced hyperthermia (Fig. 6). Interestingly, the increases in ammonia seen at 24 h after METH treatment were comparable to those previously reported at just 2 h after METH treatment (Halpin and Yamamoto 2012). These findings demonstrate that the hepatocellular damage produced by METH is sufficient to cause increases in plasma ammonia that persist for 24 h after drug exposure. Furthermore, the findings that both METH-induced liver damage and increases in ammonia were blocked by the prevention of hyperthermia further strengthen the association between METH-induced liver damage and increases in ammonia to levels that can damage the brain. This also suggests that increases in ammonia may be a mechanism by which hyperthermia contributes to METH neurotoxicity. It is worth noting that while the liver is the primary ammonia metabolizing organ, damage to other organ systems which have been reported to be altered by METH, namely the cardiac, renal, and musculoskeletal systems, also may contribute directly or indirectly to increases in ammonia (Smith and Fischer 1970; Kamijo et al. 2002; Wijetunga et al. 2003). Beyond producing increases in ammonia and contributing to METH neurotoxicity, METH-induced hepatotoxicity is likely to have other significant deleterious physiological effects. The liver is essential for the metabolism of fatty acids, cholesterol, protein, coagulation factors, and exogenous pharmaceuticals (Williams 1983; Jaeschke et al. 2002). Accordingly, acute, hepatotoxic exposure to METH has significant potential to affect any of these physiological functions and warrant further studies that examine the consequences of METH-induced hepatotoxicity. Furthermore, the extensive damage reported suggests that repeated exposure to METH has the possibility to contribute to the development of cirrhosis and potentially predispose the liver to development of hepatocellular carcinoma, as has been seen in the context of other serial, chronic insults to the liver such as viral hepatitis and alcohol exposure (Caldwell et al. 1999; Ratziu et al. 2002; McKillop and Schrum 2005).

In conclusion, these studies characterized the toxic effects of METH on the liver and demonstrated that these effects can persist for several hours after drug exposure. Furthermore, METH-induced hyperthermia was identified as a key mechanism for METH-induced hepatotoxicity and concurrent increases in peripheral ammonia. These findings further highlight the role of peripheral organ damage in the neuronal effects and toxicity of drugs of abuse and the importance of addressing hyperthermia in the clinical management of acute METH exposure.

## Abbreviations

ALT: Alanine aminotransferase
AST: Aspartate aminotransferase
H&E: Hematoxylin and Eosin
MDMA: 3,4-methylenedioxymethamphetamine
METH: Methamphetamine
Sal: Saline
